# Complete genome sequence of a putative novel orthotospovirus species identified in *Limonium sinuatum* from Colombia

**DOI:** 10.1128/mra.01263-24

**Published:** 2025-04-28

**Authors:** Marleen Botermans, Christel de Krom, Carla Oplaat, Jorik Swier, William Quaedvlieg, Annelien Roenhorst, Marcel Westenberg

**Affiliations:** 1 Netherlands Institute for Vectors, Invasive plants and Plant health (NIVIP), National Plant Protection Organization (NPPO), Netherlands Food and Consumer Product Safety Authority (NVWA), Wageningen, The Netherlands; 2 Independent Researcher, Leiden, The Netherlands; 3 Independent Researcher, Lisse, The Netherlands; Katholieke Universiteit Leuven, Leuven, Belgium

**Keywords:** orthotospovirus, novel, *Limonium*, high throughput sequencing

## Abstract

We report the complete genome sequence of a putative novel orthotospovirus species in statice (*Limonium sinuatum*) from Colombia, provisionally named Orthotospovirus limonii (Limonium orthotospovirus 1; LOV1). Its nucleocapsid protein shows less than 64% amino acid identity with other orthotospoviruses. Phylogenetic analyses place LOV1 in the “American clade.”

## ANNOUNCEMENT

Orthotospoviruses (genus *Orthotospovirus*, family *Tospoviridae*) are enveloped, quasi-spherical viruses with a tripartite RNA genome of negative and ambisense polarity, containing five open reading frames (1). These viruses are significant agricultural pathogens, causing substantial yield and quality losses in crops worldwide (2).

**Fig 1:**
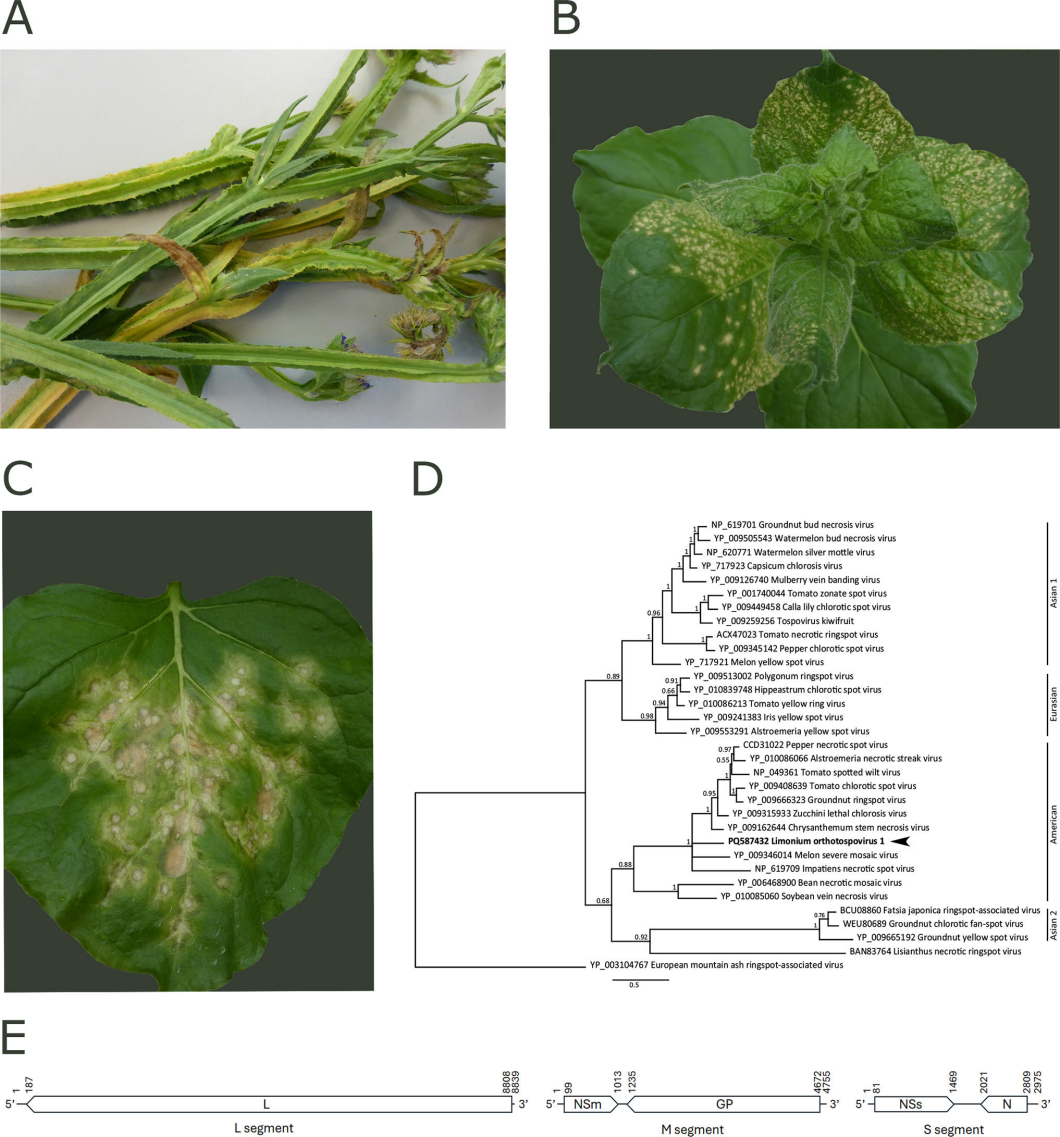
Symptoms of *Limonium sinuatum* and *Nicotiana benthamiana* infected with Limonium orthotospovirus 1, its phylogenetic relationship with other orthotospoviruses, and its genome architecture. (**A**) *L. sinuatum* exhibits mottling, chlorosis, and necrosis on leaves and stems, and a necrotic inflorescence. (**B**) *N. benthamiana* showing systemic necrotic lesions and stunted growth. (**C**) *N. benthamiana* showing local necrotic rings and lesions. (**D**) Bayesian phylogenetic tree of Orthotospovirus N protein amino acid sequences. Sequences were aligned using MAFFT (5), and the tree was constructed with MrBayes V3.2.6 (6) applying the WAG model of amino acid substitution with a gamma distribution to account for rate variation. MrBayes was run with four chains for 1.1 million generations, sampling trees every 200 generations. The first 100,000 trees were discarded as burn-ins. The tree is rooted with the European mountain ash ringspot-associated virus as an outgroup. Posterior probabilities are indicated at the nodes, and the distance bar represents 0.5 amino acid substitutions per site. Limonium orthotospovirus 1 is indicated with an arrowhead. Major clades are marked on the right. (**E**) Genome architecture of Limonium orthotospovirus 1. Nucleotide numbers are indicated at the start and end of each genome segment and predicted open reading frames. L: RNA-dependent RNA polymerase; NSm: non-structural protein; GP: glycoprotein precursor; NSs: non-structural protein; N: nucleocapsid protein.

An operator received a statice plant (*Limonium sinuatum*) from Colombia showing virus-like symptoms (Fig. 1A). A series of RT-PCR tests targeting orthotospoviruses, potexviruses, potyviruses, and tombusviruses were conducted by the operator. Only the orthotospovirus RT-PCR (3) yielded an amplicon, which was sequenced, and indicated the presence of a putative novel orthotospovirus species. Additionally, a mechanical transmission test (4) to *Nicotiana benthamiana* plants resulted in orthotospovirus-like symptoms (Fig. 1B and C). For further molecular characterization, leaf material from *N. benthamiana* was submitted to NIVIP in 2019 (NPPO-NL 39245988).

At NIVIP, high-throughput sequencing (HTS) was performed as previously described (7). In short, total RNA was extracted from 1 g of *N. benthamiana* leaf tissue using the RNeasy Plant Mini Kit (Qiagen, the Netherlands). Ribosomal RNA was depleted with the Ribo-Zero rRNA removal kit (Illumina), followed by library preparation with the NEBNext Ultra II Directional RNA Library Prep kit (New England Biolabs, USA). The library was sequenced on a NovaSeq6000 (Illumina), generating 150 nt paired-end reads.

The resulting reads (53,457,230, HQ30 >80%) were trimmed and *de novo* assembled using CLC Genomic Workbench (v21.0.4 (Qiagen) standard tools. Consensus sequences (>100 nt; read depth >10) from the *de novo* assemblies were analyzed using MegaBLAST and DIAMOND (8) with a locally installed NCBI nr(/nt) database (download 27–01-2019). Visualizations of BLAST results were carried out in Krona with a bitscore threshold of 25 (9). Only three contigs showed significant similarity to viral genomes, specifically with orthotospovirus sequences. These contigs corresponded to the three genome segments: L (8839 nt; 51,913 mean read depth [MRD]), M (4755 nt; 35,062 MRD), and S (2975 nt; 27,633 MRD) collectively encoding five open reading frames (Fig. 1E). RACE was not performed, as the conserved inverted repeats in the terminal nine nucleotides of each segment, characteristic of orthotospovirus genomes (10), were present, indicating that the genome segments were complete.

The nucleocapsid (N) protein from the S segment shared less than 64% amino acid sequence identity with other orthotospoviruses, with the highest identity (63%), observed to *Orthotospovirus chrysanthinecrocaulis* (Chrysenthemum stem necrosis virus; CSNV). This level of identity is well below the 90% species demarcation threshold established for the genus *Orthotospovirus* (1), supporting the designation of this virus as a novel orthotospovirus species, provisionally named Orthotospovirus limonii (Limonium orthotospovirus 1; LOV1). To gain insights into the phylogenetic placement of the N protein sequence, a Bayesian tree was inferred using Geneious Prime 2023.1.1 (Biomatters, New Zealand). The resulting tree positioned LOV1 within the “American clade” (Fig. 1D).

Since HTS was conducted on a test plant rather than the original host, the presence of other viruses remains unknown. Additionally, the role of physiological or environmental factors in symptom development is unclear. Further biological and epidemiological studies are needed to expand knowledge of this virus, its potential thrips vector, and its impact on crops.

## Data Availability

The complete genome sequence has been deposited at GenBank under accession numbers PQ587430, PQ587431, and PQ587432. The raw reads were deposited under SRA accession number SRR31333954.

## AFTER EPUB

[This article was published on 28 April 2025 with an error in the affiliations. The affiliations were corrected in the current version, posted on 29 May 2024.]
